# Low Concentration of Antibiotics Modulates Gut Microbiota at Different Levels in Pre-Weaning Dairy Calves

**DOI:** 10.3390/microorganisms6040118

**Published:** 2018-11-27

**Authors:** Mohammed Husien Yousif, Jing-Hui Li, Zheng-Qian Li, Gibson Maswayi Alugongo, Shou-Kun Ji, Yuan-Xiao Li, Ya-Jing Wang, Sheng-Li Li, Zhi-Jun Cao

**Affiliations:** 1 State Key Laboratory of Animal Nutrition, College of Animal Science and Technology, China Agricultural University, Beijing 100193, China; mohammedago@hotmail.com (M.H.Y.); lgreyh@hotmail.com (J.-H.L.); maswayi@yahoo.com (G.M.A.); jishoukun@163.com (S.-K.J.); yajingwang@cau.edu.cn (Y.-J.W.); lisheng0677@163.com (S.-L.L.); 2 College of Animal Science and Technology, Henan University of Science and Technology, Luoyang 471003, China; 18238839603@139.com (Z.-Q.L.); lyx8032@yeah.net (Y.-X.L.)

**Keywords:** ileum, colon, rectum, calf, antibiotics, microbiota

## Abstract

The aim of this study was to investigate the effect of feeding milk replacer (MR) with two different antibiotics treatments on the gut microbiota of pre-weaning calves. Twelve (12) Holstein male calves at 1-day-old were randomly assigned to: milk replacer without antibiotics (CON), milk replacer plus low cocktail of antibiotics (LCA) concentration (penicillin 0.024 mg/L, streptomycin 0.025 mg/L, tetracycline 0.1 mg/L, ceftiofur 0.33 mg/L), and milk replacer plus a low concentration of single antibiotic (LSA; ceftiofur 0.33 mg/L). All the calves were harvested at 35-day-old, and the digesta from the ileum and colon was collected in addition to fecal samples. Samples were analyzed by 16S rRNA gene using Illumina MiSeq platform. Results showed that there were significant differences among treatments in the ileum, where LCA significantly reduced the relative abundance of Enterobacteriaceae (*P* = 0.02) especially *Escherichia-coli* (*P* = 0.02), while LSA significantly reduced the relative abundance of *Comamonas* (*P* = 0.02). In the colon and rectum, LSA treatment was significantly enriched with the class *Bacilli*, whereas the control group was significantly enriched with *Alloprevotlla* (*P* = 0.03). However, at the family level in the rectum LCA and LSA significantly reduced the relative abundance of Acidaminococcaceae (*P* = 0.01). Moreover, at the genera level in the colon, LSA significantly increased *Prevotellaceae_Ga6A1_ group* (*P* = 0.02), whereas in the rectum both of treatments reduced the relative abundance of *Phascolarctobacterium* (*P* = 0.01). In conclusion, the overall low cocktail of antibiotics concentration induced changes at different taxonomic levels; specifically the decrease in *Escherichia-coli* which might subsequently reduce the incidences of diarrhea in calves.

## 1. Introduction

The gastrointestinal tract (GIT) of a young calf is colonized by millions of microbiota immediately after birth [1,2]. These microbiota play an indispensable role in the host by extracting energy and other essential nutrients and development of the gut tissues and immune system [1,3,4]. Balanced intestinal microbiota is fundamental for healthy calves that allow them to grow adequately [5]. The pre-weaning period is considered as a critical window, in which the calf microbiota is established [1,2]. Various external factors such as the diet and use of antibiotics can significantly influence the establishment of these microbiota [1,6]. On most dairy farms in the world calves are either fed waste whole milk or milk replacers (MR). The waste milk encompasses milk from fresh cows and those cows that are undergoing treatment with antibiotics [7]. Furthermore, the waste milk may contain a high amount of somatic cell counts and pathogenic microorganisms [7,8]. Since the antibiotics can be passed through milk, calves fed on milk from cows undergoing treatment are likely to consume a substantive amount of antibiotics. Similarly, calves can be fed a medicated MR, which is a MR that has been supplemented with oxytetracycline and neomycin, at a ratio of 1:1, concentration fed at either a low (0.11 to 0.22 mg/kg) or high (22 mg/kg) [9] and generally for prevention and as growth promoters [10]. Few studies have attempted to investigate the effect of feeding waste milk on young calves, although the use of antibiotics in livestock is a controversial issue due to public health concerns. In a recent study, Deng et al. [11] showed that waste milk pre-treatment significantly affected the microbiota composition of pre-weaning calves at genera level. Calves that were fed with medicated MR reported reduced morbidity and calf scours compared to those fed non-medicated MR [12,13,14]. This reduction was likely due to modulation of the intestinal and fecal microbiota in the pre-weaning period [15,16,17]. Xie et al. [16] and Looft et al. [18] reported that in-feed antibiotics led to an increase in the abundance of pathogenic microbes like *Escherichia-coli* in calves and pigs, respectively. *Escherichia-coli* is the most abundant facultative anaerobic species in the gastrointestinal tract of human and animals, usually a commensal microbe, but it is also of great importance since it gives rise to several diseases in humans and animals [19,20]. Yu et al. [21] showed a decrease in the relative abundance of Enterobacteriaceae in the cecum digesta of weanling pigs when they were fed with basal diet supplemented with polymyxin, low-molecular-weight chitosan, and zinc oxide. Reports at different taxonomic levels vary between studies. Pereira et al. [15] reported that feeding milk with drug residues induced changes at the genus level only, and decreased the relative abundance of *Clostridium* and *Streptococcus*. In a piglets study, the diet that contained Zinc oxide or antibiotics (chlortetracycline and colistin sulfate) significantly increased five phyla Spirochaetes, Tenericutes, Euryarchaeota, Verrucomicrobia, TM7, and reduced one phylum (Chlamydiae) in the ileal digesta [22]. Furthermore, the microbial diversity of ileal digesta increased due to the addition of Zinc oxide or antibiotics, while that of colonic digesta decreased [22]. The inconsistent findings on the effect of antibiotics on fecal microbiota are possibly due to the kind of antibiotics, dose of antibiotics, different animals, and different kind of samples.

Studies concerning the impact of antibiotics on pre-weaning calves’ microbiota have focused mainly on fecal microbiota because of the difficult and/or cost of sampling the rest of the gut. Nonetheless, the changes in the bacterial composition and activity might differ between the small intestine and the hindgut [23]. To the best of our knowledge, there is no research on the effect of ingesting a low concentration of antibiotics via MR on the gut microbiota of pre-weaning calves. We hypothesized that ingestion of low concentration of antibiotics through waste milk could induce changes in the gut microbiota of pre-weaning calves. We hypothesized that ingestion of low concentration of antibiotics through waste milk could induce changes in the gut microbiota of pre-weaning calves. Since antibiotics residues in the waste milk varied to a large extent we tried to adjust the level of antibiotics concentration which is conveniently prepared via MR; therefore, we mimicked the amount of the most frequently used antibiotics in a dairy farm fed with MR. This study aimed to investigate the impact of supplementing low doses of antibiotics with MR on gut microbiota of the pre-weaning calves and postulated to what extent the changes in the microbiota are either beneficial or harmful to the pre-weaning calves. 

## 2. Materials and methods

### 2.1. Animals, Diets and Sample Collection

A total of 12 new-born male Holstein dairy calves were included in this experiment during the period from October to December 2016 that had an initial body weight of 41.08 ± 2.71 kg. All calves were fed 4 L of maternal colostrum obtained within 1 hr after birth. They were then moved to individual hutches lined with straw bedding on day 2 and offered free access to water and starter feed from day 4. The starter used in this study to feed the calves was free from any kind of antibiotics. Twelve calves were selected from cows that had never received antimicrobial treatments during the transition period before calving. Calves were weighed, and a sample of blood from the jugular vein was used to estimate plasma protein score by refractometer to assess the adequacy of immunoglobulin passive transfer (plasma protein ≥ 5.6 g/dL). Concentration and selection of antibiotics were based on the most frequently used medicines in the SUNLUN Livestock, Dingzhou (Hebei, China), dairy farm. Over a 3-week period, 3 samples of milk were collected for each week, a total of 9 milk samples to test the antibiotics residual. We used 4 enzyme-linked immunosorbent assay (ELISA) kits from Beijing WDWK Biotechnology Co., Ltd, China, to test the amount of antibiotics residues in the waste milk on this farm (penicillin, streptomycin, tetracycline and ceftiofur; Table S1). The antibiotic residues varied to a large extent. Thus, we used the median amount of the antibiotics in this study. Additionally, ceftiofur had the highest concentration of all the antibiotics added. Likewise, among the antibiotics added to the non-saleable raw milk in the Pereira et al. [24] study, Ceftiofur had the highest concentration. Milk replacer was provided two times per day with the amount of MR varying depending on the age of the calf (2 L/meal up to day 5, 3 L/meal from day 6 to 14 and 4 L/meal from day 15 to 34). Mobile stainless tanks (120–200 L, MilchMobile 4 × 4 from Forster-Technik) were used to distribute the MR at 7:30 and 16:00. The experimental design and procedures were executed according to the protocols approved by the Ethical Committee of the College of Animal Science and Technology, China Agricultural University 10/3/2016 (protocol number. 2016DR07).

The calves were randomly assigned to each of the three treatments; calves were fed a MR without antibiotics (CON), a MR plus a low cocktail of commercial antibiotics concentration (LCA; 0.024 mg/L penicillin, 0.025 mg/L streptomycin, 0.1 mg/L tetracycline and 0.33 mg/L ceftiofur) and a MR plus a low concentration of single commercial antibiotic (LSA; 0.33 mg/L ceftiofur). Antibiotics were not used to treat the calves suffering from diarrhea or any health problems during the experimental period. Calves that suffered from diarrhea were given electrolyte solution. To assure that no contamination between calves, each calf stall had devoted utensils and supplies, and disposable clothes were used during sample collection to avoid cross-contamination between samples. The nutrient composition of the MR (Friesland Campina, Netherlands) used in this study is as shown in Table 1. No antibiotics were added to the MR or calf starter.

A solution for each of the antibiotics was prepared separately to avoid any interaction between the drugs. The solutions were prepared every three days and added to an individual bucket of milk as per the treatment using an automatic pipette (Eppendorf) and disposable tips. The antibiotics were mixed thoroughly with the MR and the calves were fed immediately after mixing. 

On day 35 fecal samples were collected rectally using disposable gloves from each calf and stored at −20 °C for further analysis and subsequently four calves per treatment were harvested for the collection of hindgut digesta. Euthanasia was executed by injecting 1 mL of xylazine into the calf intravenously. The esophagus and rectum were first tied to prevent the gastrointestinal tract GIT from cross-contamination and to keep the content inside GIT following euthanasia. The other gut sections including the ileum and colon segments were identified and isolated using thread to keep the gut content of each section separately. The digesta were collected from the ileum and colon immediately after harvesting and were snap-frozen in liquid nitrogen and stored at −20 °C until further analysis. 

### 2.2. DNA Extraction and Polymerase Chain Reaction (PCR) Amplification 

Metagenomic DNA was extracted from each of the 36 samples from ileum, colon and rectum using DNA Stool Mini Kit (Qiagen, Hilden, Germany) according to the manufacturer’s instructions. The V3-V4 region of the bacterial 16S rRNA genes was amplified by PCR, using specific barcode primers 338 F (5′-ACTCCTACGGGAGGCAGCAG-3′) and 806 R (5′-GGACTACHVGGGTWTCTAAT-3′) as described by Bi et al., [25]. The PCR was carried out by the following protocol: an initial denaturation step at 95 °C for 2 min, followed by 30 cycles at 95 °C for 30 s, annealing at 55 °C for 30 s, and extension at 72 °C for 30 s and a final extension at 72 °C for 5 min. The PCR products were visualized on 2% agarose gels and were quantitatively determined using QuantiFluor-ST Fluoremeter (Promega, Wisconsin, USA) and PicoGreen dsDNA Quantitation Reagent (Invitrogen, Carlsbad, CA, USA). The PCR amplicons were purified with an AxyPrep DNA Purification kit (Axygen Biosciences, Union City, CA, USA).

### 2.3. Illumina MiSeq Next generation Sequencing. 

Purified amplicons were pooled in equimolar and paired-end sequenced (2 × 250) libraries of Illumina MiSeq® platform at Beijing Allwegene Technology Co., Ltd, China. 

### 2.4. Data Processing and Bioinformatics Analysis.

After trimming the adaptor and primer sequences from Illumina reads, the raw sequences were collected for each sample according to a unique barcode of the Quantitative Insight Into Microbial Ecology (QIIME) (v.1.17; Quantitative Insight into Microbial Ecology). The following standards were applied: (i) the 300-bp reads were truncated at any site that obtained an average quality score of <20 over a 10-bp sliding window and the truncated reads shorter than 50 bp were discarded; (ii) exact barcode matching, two nucleotide mismatch in primer matching, and reads containing ambiguous characters were removed; (iii) only overlapping sequences longer than 10 bp were assembled according to their overlapped sequence. Operational taxonomic units (OTUs) with 97% similarity cut-off were assembled using UPARSE [26], and chimeric sequences were identified and removed using USEARCH (v.6.1) [27]. The representative sequences were aligned against the Silva release 119 gene database and classified by ribosomal database project (RDP) classifier (v.2.2) [28]. The rarefaction analysis based on Mothur (v.1.21.1) was conducted to reveal the diversity indices. The alpha diversity for each sample was performed to estimate Chao1, Shannon, and observed species, using the QIIME pipeline (v.1.5.0) [27]. The relative abundances of main phyla and family were calculated with average > 0.1%, and the main relative abundances of the genera were estimated with average abundance >0.05%. The high-throughput sequencing data generated were uploaded to the Genome Sequence Archive, (Genomic, Proteomics and Bioinformatics 2017) in Big Data Center, www.bigd.big.ac.cn/gsa, under the accession number (CRA000901). 

### 2.5. Statistical Analysis

The relative abundance and significant differences in the bacterial community were assessed by linear discriminant analysis (LDA) effect size with the Kruskal–Wallis test and the LDA score (log 10) >3.5 for all comparisons and any taxa should be represented in at least 60% of the total samples. Principal components analysis (PCA) using OTUs from each sample regarding the Euclidean distance between samples was calculated according to Wang et al. [29]. One-way analysis of variance (ANOVA) with Bonferroni in GraphPad Prism software (version 5, GraphPad Software, La Jolla California USA) was used to compare the relative abundances of the main phyla, families, and genera. 

## 3. Results

### 3.1. Characteristics of Sequenced Data

The high throughput sequencing of bacterial DNA generated 1,046,951 raw reads. After data quality filtering and chimera removal, valid sequences based on 97% similarity were obtained for the ileum (26393 ± 8537), colon (30435 ± 2207), and rectum (23117 ± 8394). The overall numbers of OTUs assigned were 249.67 ± 40.91, 187.57 ± 20.29, and 181.42 ± 34.45 for the ileum, colon, and rectum, respectively. Any taxa found to be ubiquitous in at least 60% of samples was defined as a core microbiome. Thus, ileum, colon, and rectum had 35, 50 and 41 shared OTUs among treatments, respectively (Figure S1).

All samples reached a stable plateau and a reasonable asymptote based on rarefaction curves analysis, suggesting that the samples provided sufficient depth to describe the bacterial communities. (Figure S2) 

### 3.2. Alpha Diversity of Microbiota in the Ileum, Colon, and Rectum

The alpha indices (Shannon, Chao 1, and observed species) did not differ significantly (*P* < 0.05) among the treatments in the ileum and colon (Table 2). However, in the rectum, significant differences in Chao1 (*P* = 0.002) and observed species (*P* = 0.01) were found with the CON recording significantly higher values than LSA treatment (*P* < 0.05; Table 2). Generally, the results showed that LCA had relatively higher bacterial richness (Chao 1, and observed species) and bacterial diversity (Shannon) in the ileum and colon though not significant.

### 3.3. Principle Component Analysis (PCA) for Beta Diversity 

**Figure 1:**
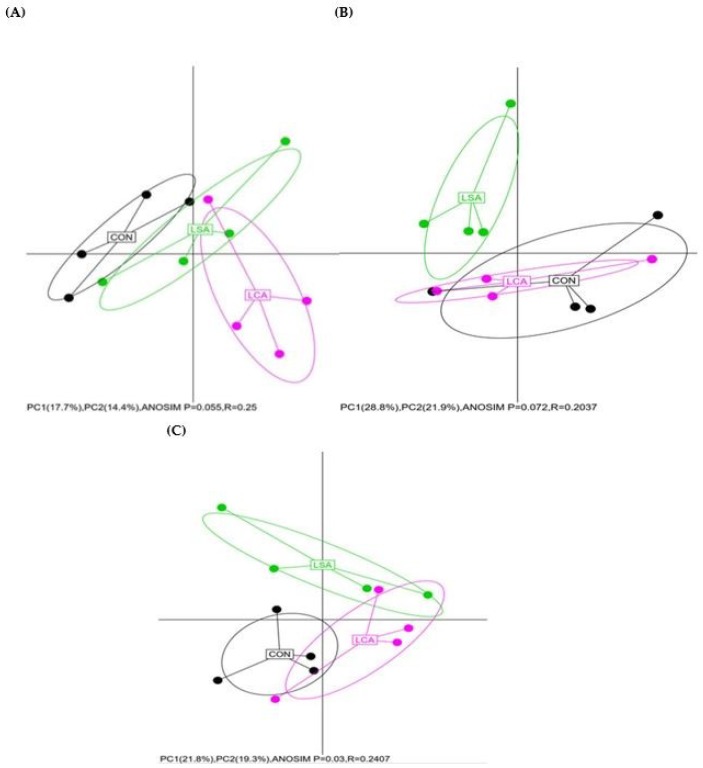
Principal component analysis (PCA) of bacterial community structures in the three treatments of the ileum (**A**), colon (**B**) and rectum (**C**). The percentage of variation explained by the x axis represented by PC1 and the y axis represented by PC2 are indicated in the axis. A = ileum; B = colon and C = rectum. CON = control; LCA = low cocktail of antibiotics concentration; LSA = low concentration of single antibiotic. The percentage of variation explained by PC1 and PC2 are indicated in the axis, and the ellipses represented with 95% confidence. R-values closer to 0 denote groups not significantly different from one another, while values closer to 1 denote a highly different community composition, *P* value ≤ 0.05 is significantly different.

We used PCA for beta diversity assessment, a metric that takes into account the phylogenetic divergence between OTUs, in order to assess the differences among the ileal, colonic and rectal microbial communities by treatment. The PCA indicated distinct clustering and significant difference among treatments in the rectum (*P* = 0.03; Figure 1C), while in the colon CON and LCA showed some similarities and the LSA separated from CON and LCA (Figure 1B). In the ileum, there was a clear tendency to clusters according to the treatment, although the difference is not significant among treatments (Figure 1A).

### 3.4. Microbiota Composition of the Ileum, Colon and Rectum

**Figure 2:**
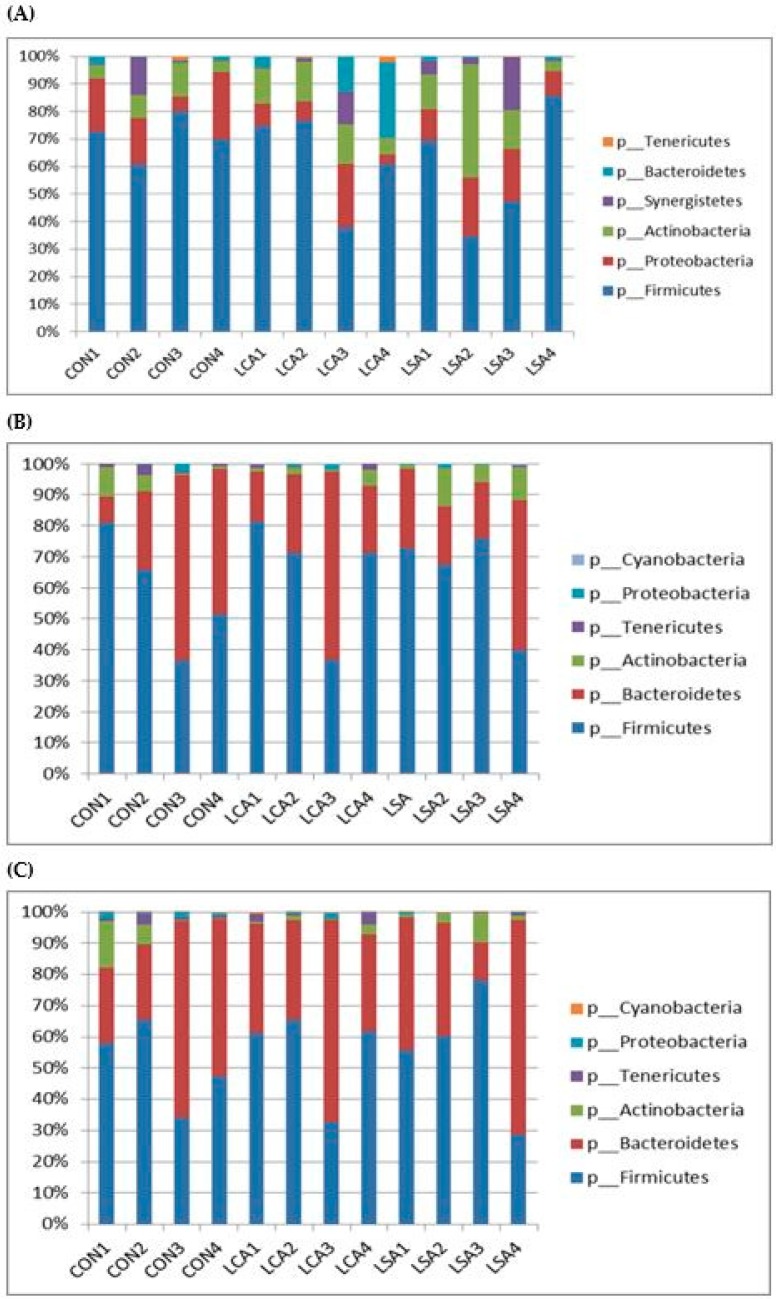
Effect of feeding milk replacer (MR) with low antibiotics concentration cocktail or single on the phyla composition in the ileum, colon and rectum. A = ileum; B = colon and C = rectum. CON = control; LCA = low cocktail of antibiotics concentration; LSA = low concentration of single antibiotic. The values are the mean plus standard deviation.

At phyla level, 14 phyla were identified with higher mean as predominant in the ileum, while in the colon and rectum 11 phyla were identified in each section (Figure 2A–C). The top five predominant phyla in the ileal content were Firmicutes, Proteobacteria, Actinobacteria, Synergistetes, Bacteroidetes with Firmicutes and Proteobacteria making up over 70% of the sequences. On the other hand, Firmicutes, Bacteroidetes, Actinobacteria, Tenericutes, and Proteobacteria were the predominant phyla in the colon and rectum. The results of the current study showed a higher relative abundance of Firmicutes in the ileum in CON, while in the colon the relative abundance of *Firmicutes* in the antibiotics treatments was higher than relative abundance in the corresponding treatments of the ileum and rectum. On the other hand, Proteobacteria displayed a higher relative abundance in the ileum across all treatments compared to the colon and rectum. Similarly, Bacteroidetes in the rectum showed higher relative abundance across all treatments compared to the ileum and colon (Table S2). For other minor phyla such as Actinobacteria and Cyanobacteria, showed a higher relative abundance in the ileum compared to colon and rectum, whereas the relative abundance of Tenericutes was higher in the rectum compared to the ileum and colon. However, there were no significant differences detected in the predominant phyla among the different treatments in the three sections indicating that the low concentration of antibiotics did not induce changes at the phylum level (Table S2).

**Figure 3:**
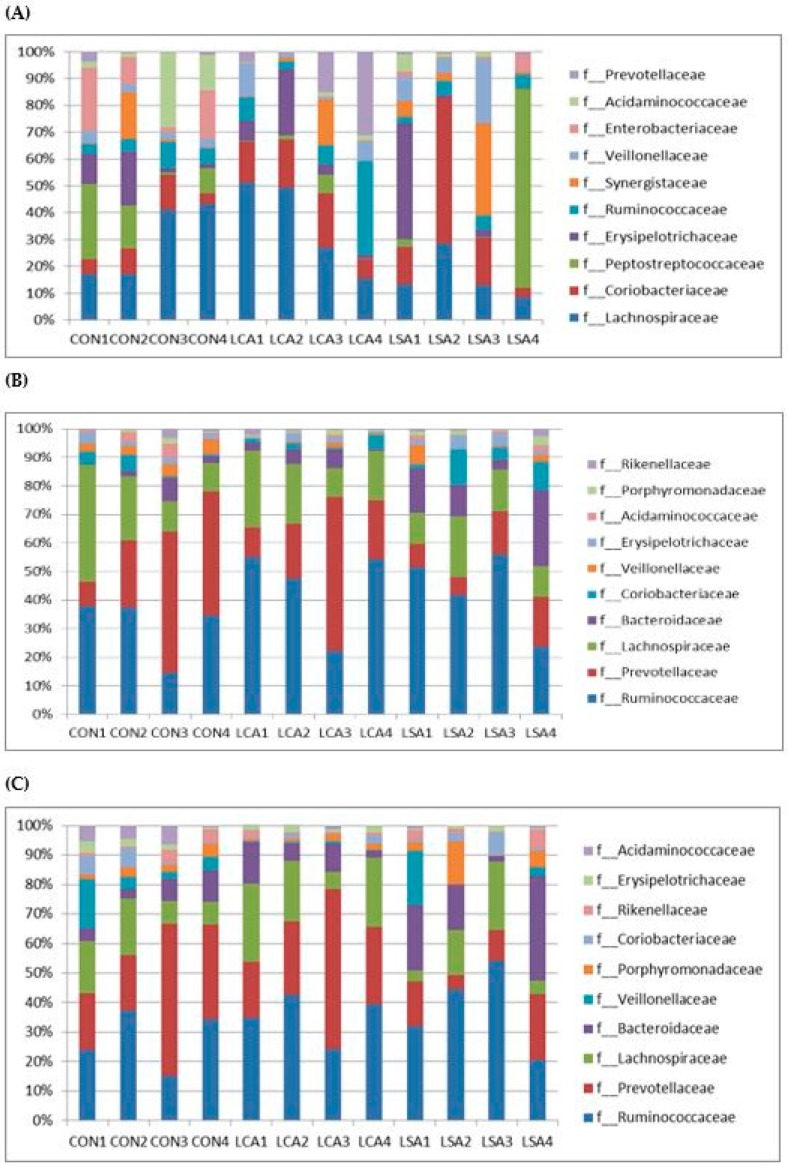
Effect of feeding MR with low antibiotics concentration cocktail or single on the family distribution in the ileum, colon and rectum. A = ileum; B = colon and C = rectum. CON = control; LCA = low cocktail of antibiotics concentration; LSA = low concentration of single antibiotic. The values are the mean plus standard deviation.

Lachnospiraceae, Coriobacteriaceae, Peptostreptococcaceae, and Erysipelotrichaceae were the most abundant families in the ileum (Figure 3A), with Lachnospiraceae, being the most predominant family in CON (0.2343 ± 0.12) and LCA (0.2717 ± 0.15), whereas Peptostreptococcaceae (0.1775 ± 0.34) was the most predominant family in LSA (Table S3). However, LCA significantly reduced the relative abundance of Enterobacteriaceae (*P* = 0.02) compared to CON (*P* = 0.02). In the colon and rectum, the predominant families were Ruminococcaceae, Prevotellaceae, Lachnospiraceae, and Bacteroidaceae (Figure 3B,C). Prevotellaceae (0.2969 ± 0.18) was the most abundant family in CON, while Ruminococcaceae was more abundant in LCA (0.4357 ± 0.15), and LSA (0.3694 ± 0.14; Table S3). Results showed an increase in the relative abundance of Bacteroidaceae in LSA, as well as a decrease in the relative abundance of Prevotellaceae in the colon (Table S3). However, LCA and LSA significantly reduced the relative abundance of Acidaminococcaceae (*P* = 0.02) in the rectum. 

**Figure 4:**
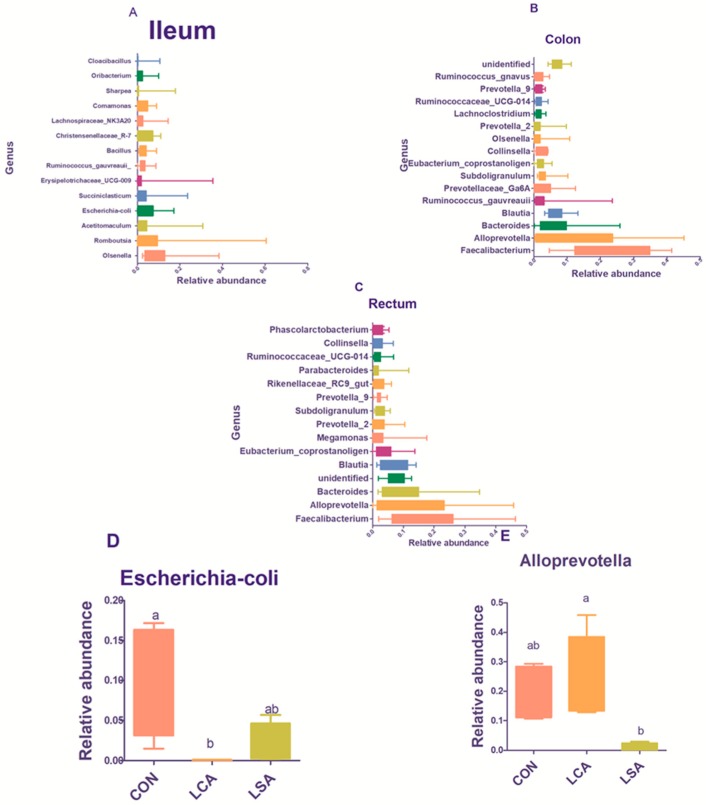
Effect of feeding MR antibiotics on the predominant genera in the ileum, colon and rectum samples. A = ileum; B = colon; C = rectum; D and E Comparison of the relative abundances of the significantly different genera among treatments in the ileum and rectum respectively. CON = control; LCA = low cocktail of antibiotics concentration; LSA = low concentration of single antibiotic. The values are the mean plus standard deviation. Treatments with different letters are significantly different (*P* < 0.05).

At the genus level, 217, 138, and 132 genera were identified, in the ileum, colon, and rectum respectively (Figure 4A–C). These included 96, 70, and 61 genera identified as core microbiome in the ileum, colon, and rectum respectively. The most abundant genera in the ileum were *Olsenella*, *Romboutsia, Acetitomaculum,* and *Escherichia-coli* (Table S2; Figure 4A). While in the colon and rectum the most abundant genera and most common between the two sections were *Faecalibacterium, Alloprevotella, Bacteroides, Blautia* (Table S2). The other predominant genera in the colon were *Ruminococcus_gauvreauii, Subdoligranulum,* and *Prevotellaceae_Ga6A1_ group*. The other dominant genera in the rectum were *Eubacterium* and *Megamonas* (Table S2). The study showed that LCA significantly reduced the relative abundance of *Escherichia coli* (Table S2; Figure 4D) and increased the relative abundance of *Faecalibacterium*, *Alloprevotella*, and *Bacillus* in the ileum. On the other hand, LSA significantly increased the relative abundance of *Comamonas* (*P* = 0.02). Furthermore, a slight increase was observed in the relative abundance of *Romboutsia* and a simultaneous decrease in the relative abundance of *Streptococcus* (Table S2). In the colon and rectum generally, the LCA increased the relative abundance of *Faecalibacterium, Alloprevotella, Blautia,* and *Bacteroides*. Similarly, LSA increased the relative abundance of the same genera in the rectum except for the *Alloprevotella* which was significantly reduced (*P* = 0.03; Table S2; Figure 4E). However, in the colon, the LSA significantly increased the relative abundance of the *Prevotellaceae_Ga6A1*_ group (*P* = 0.02), while in the rectum LCA and LSA significantly decreased the relative abundance of *Phascolarctobacterium* (*P* = 0.01; Table S2).

**Figure 5:**
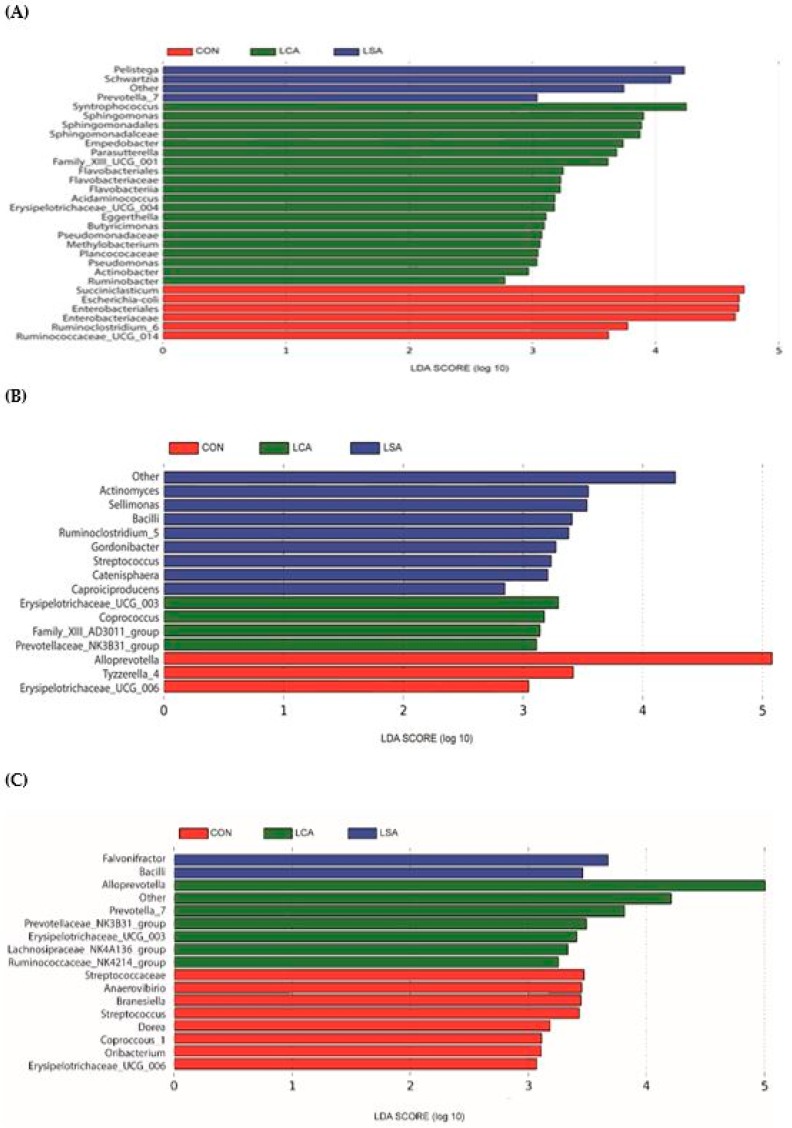
Linear discriminant analysis (LDA) for the significantly abundant microbes across treatments in the ileum, colon and rectum. Histogram of the LDA scores calculated for differentially abundant genera in the ileum, colon and rectum at the genus level among the different treatments groups (only the genera LDA scores above 2 are shown). A = ileum; B = colon and C = rectum. CON = control; LCA = low cocktail of antibiotics concentration; LSA = low concentration of single antibiotic.

We used LEfSe to further identify the taxa that were significantly different in the ileum, colon and rectum (Figure 5A–C). Noteworthy, changes in the ileal microbiota at different taxonomic levels were found across the different treatments. Enterobacteriales and Enterobacteriaceae with three genera, *Escherichia-coli, Ruminococcaceae_UCG-014,* and *Succiniclasticum,* significantly enriched in CON, whereas LCA had 3 genera, *Syntrophococcus*, *Empedobacter*, and *Parasutterella* (Figure 5A). The LSA treatment showed a higher relative abundance for two genera of *Pelistega* and *Schwartzia*. The colonic content of CON was significantly enriched with *Alloprevotella* and *Tyzzerella-4* (*P* < 0.05; Figure 5B). On the other hand, LSA was significantly enriched with *Bacilli* in addition to *Actinomyces* and *Sellimonas*. There were 8 bacterial taxa that were significantly enriched in all treatments in the rectum, with the CON (*P* < 0.05; Figure 5C) having two genera *Anaerovibrio*, and *Branesiella*. The LSA was different from CON in class *Bacilli* (*P* < 0.05), and genus *Flavonifractor*. The LCA was significantly enriched with five genera (*Alloprevotella*, *Prevotella_7*, *Prevotellaceae _ NK3B31 _ group*, *Lachnospiraceae_NK4A136_group*, and others). 

## 4. Discussion

The importance of using antibiotics to enhance the performance of young calves is unprecedented in literature. On the other hand, gastrointestinal microbiota is a major crux in the health and performance of a pre-weaning calf. Herein, we observed that low concentration of antibiotics could induce changes in the gut microbiota of pre-weaning calves. Similar controlled studies are scarce, although attempts have been made with fecal microbiota [15,16,17] to depict the role of antibiotics in the gut. In rectal samples, Chao1 and observed species were higher in CON than LSA, which was similar to previous studies that used fecal samples [17,24,30]. The dominant phyla in the ileum, colon, and rectal samples, regardless of treatment, were Firmicutes, Bacteroidetes, and Proteobacteria consistent with previous research [30,31,32]. Pereira et al. [15] showed that antibiotics affected the microbiota at the genus level only; while our results showed that ingestion of antibiotics induced a shift at higher bacterial taxonomic levels. This might imply that fecal samples cannot comprehensively have an unequivocal effect of antibiotics on hindgut microbiota. 

Dysbiosis is the main cause of diarrhea in the pre-weaning calves. In his review Constable [10] showed that *Escherichia-coli* is usually high in the hindgut, but concurrently increases with higher incidences of diarrhea in the ileum. Family Enterobacteriaceae tends to have more potential pathogenic bacteria such as genus *Escherichia-coli* [33] which is a primary initiator of diarrhea in calves. The LCA group exhibited a decrease in the abundance of both Enterobacteriaceae and *Escherichia-coli* in the ileum. The results were corroborated by LDA analysis that showed higher *Escherichia-coli* in the CON group. Similarly, Yu et al. [21] reported a significant reduction in the family Enterobacteriaceae in the cecal samples of weaned piglets when fed on a diet supplemented with antibiotics. In contrast to our results, Xie et al. [16] reported that treating pre-weaning calves with a broad spectrum antibiotic, bacitracin methylene disalicylate (BMD) increased the prevalence of *Escherichia-coli* in the fecal microbiota. The reasons behind these differences are unclear but are likely related to different antibiotics or the type of samples used. Thus, we speculated that LCA might affect the establishment of the microbiota at an early age and simultaneously improve the health of the calves by significantly reducing *Escherichia-coli* and subsequently reducing diarrhea. Moreover, sufficient concentration of antibiotics in the milk feed is likely to select for resistant *Escherichia-coli* in the gut [34]. This might partly explain the lack of differences between calves fed Ceftiofur only and calves not receiving antibiotics in MR. In a recent study, Duse et al. [35] postulated that an increase in quinolone-resistant *Escherichia-coli* on farms in Sweden that were utilizing penicillin might have been as a result of this antibiotic clearing penicillin-susceptible bacteria in the gut of the calves surveyed. However, similar observations were not made in the colon and rectum samples of our study. 

The LDA analysis further revealed that reduction in *Escherichia-coli* resulted in enrichment of other taxa in the ileum. The importance of these microbiota in calves receiving antibiotics have not been well articulated, maybe due to their low abundance in the gut and which is beyond the scope of this study. Furthermore, it was not possible to determine whether the increase is due to resistance to the classes of antibiotics or reduction in *Escherichia-coli*. It is worth noting that some of the bacteria such as *Comamonas* [31], *Pseudomonas* [36] and *Syntrophococcus* [2] have been previously reported to be present in the gut of calves. Oikonomou et al. [30] reported a higher prevalence of *Comamonas* in the fecal matter of pre-weaning calves. The identification of this genus in different GIT locations raises more questions about its role in the GIT. The reasons underlying the increase in this genus in LSA treatment are not clear, and further studies are recommended. Derakhshani et al. [36] reported that *Pseudomonas* was negatively correlated with mucosal inflammation, implying that these species might not elicit immune responses in the gut. Furthermore, differences can be due to different factors like the environment, type of feeding, husbandry practices and the use of antibiotics. Gomez et al. [37] reported a difference in the relative abundance of Acidaminococcaceae in healthy calves between two farms. In this study, LSA or LCA had significantly reduced the relative abundance of Acidaminococcaceae compared to the CON. On the other hand, the genus *Phascolarctobacterium*, which produces propionic acid by fermentation of succinate [38] was significantly higher in CON. Antibiotics in LCA and LSA treatments might have exerted selective pressure on some bacterial species that produce succinate and subsequently affecting the prevalence of *Phascolarctobacterium*. Interestingly, the relative abundance of *Faecalibacterium* and *Alloprevotella* relatively increased in the LCA treatment, whereas the relative abundance of *Streptococcus* decreased. Our study is in accordance with the findings of Pereira et al. [15] on pre-weaning calves which elaborated the decrease in *Streptococcus* genus in calves received milk with drug residues. Therefore, we speculated that antibiotics might have upset the presence of *Streptococcus* in the GIT. Moreover, the association between weight gain and the abundance of *Faecalibacterium* has been demonstrated [30]. 

Generally, many of the genera in the family Prevotellaceae (*Alloprevotella*, *Prevotella_7*, and *Prevotellaceae_NK3B31*) were enriched in the rectum of the LCA group. Specifically, the relative abundance of *Alloprevotella* was low in LSA in both the colon and the rectum, but much higher in LCA of the rectum and CON of the colon. These results showed that a concoction of several antibiotics was effective in maintaining the population of *Alloprevotella* in the hindgut compared to using Ceftiofur alone. *Alloprevotella* mainly produces succinate and acetate as end products in the gut [39] which might imply that this species was abundant due to the presence of undigested fiber in the hindgut. Dou et al. [40] showed a strong association between Prevotellaceae and health in post-weaning diarrheic piglets. Zhang et al. [41] reported a high prevalence of the genus *Prevotella* in the gut of piglets due to resistant chlortetracycline treatment. Kim et al. [42] documented that the increase in relative abundance of *Prevotella* due to antibiotics administration might contribute to the development and maturation of specific microbiota in the gut of the piglets.

Meanwhile, most of the OTUs in the LSA group belonged to *Bacilli*, similar to Oikonomou et al. [30] who found that *Lactobacillus* spp. were at maximum during the fourth week of life in calves. Higher abundance of *Bacilli* and *Streptococcus* were recently reported in the gut of mice prepared with antibiotics for fecal microbiota transplantation [43]. Our findings support an earlier study by Rafii et al. [44] and his colleagues who revealed the ability of some *Bacillus* strains to grow in the presence of ceftiofur and to degrade ceftiofur to compounds that could not be examined by HPLC and lacked bactericidal activity. *Bacilli* tend to have some species that can degrade ceftiofur, and produce ceftiofur-degrading β-lactamases enzymes in the large intestines of cattle [45]. Therefore, further characterization of β-lactamases enzymes isolated from *Bacilli* in the LSA might help to further assess whether these enzymes are reservoirs for antimicrobial-resistance genes that could be transferred to the pathogenic bacteria.

## 5. Conclusions

Our study showed that low concentrations of antibiotics had differential effect at different levels (class, order, family, and genus) in the gut microbiota of pre-weaning calves. Moreover, the use of LCA was more effective than LSA, considering the significant reduction in *Escherichia-coli,* which might be taken as a good indicator of a healthy gut. However, *Escherichia-coli* is a commensal genus that plays a pivotal role in the sequential establishment of the gastrointestinal microbiota. Further studies to determine precisely the effect of antibiotics on the gut microbial establishment and the benefits of antibiotics in early life are required.

## Figures and Tables

**Table 1:** Nutrient composition of milk replacer and starter.

Nutrient	Milk Replacer	Starter
Dry matter(DM), %	96.0	89.1
CP%, DM	22.0	25.6
EE%, DM	20.0	3.9
Ash%, DM	9.0	7.9
Ca%, DM	0.6	0.6
P%, DM	0.6	1.6
Lactose%, DM	39.7	—
NDF%, DM	—	24.5
ADF%, DM	—	8.4

CP = crude protein, EE = ether extract, NDF = neutral detergent fiber, ADF = acid detergent fiber.

**Table 2:** The effect of low amount of antibiotics on operational taxonomic units (OTUs) and alpha diversity for ileum, colon and rectum.

Ileum	CON	LCA	LSA	*P* Value
OTUs	248.5 ± 17.3	259.3 ± 59.0	241.3 ± 46.2	0.87
Chao1	255.4 ± 17.6	272.0 ± 53.6	260.7 ± 33.4	0.84
Observed species	199.8 ± 26.3	220.9 ± 49.0	196.8 ± 39.8	0.71
Shannon	4.84 ± 0.21	5.07 ± 0.55	4.13 ± 1.00	0.27
Colon				
OTUs	186.5 ± 22.8	196.5 ± 16.2	180.3 ± 23.4	0.61
Chao1	206.4 ± 20.4	214.0 ± 15.0	196.7 ± 20.6	0.49
Observed species	181.2 ± 23.1	188.5 ± 13.8	173.8 ± 25.9	0.70
Shannon	4.49 ± 0.6	4.39 ± 0.5	4.68 ± 0.6	0.81
Rectum				
OTUs	218.5 ± 14.2 ^a^	184.0 ± 8.2 ^b^	141.8 ± 12 ^c^	0.0003
Chao1	223.7 ± 17.6 ^a^	208.7 ± 10.5 ^a^	157.8 ± 8.7 ^b^	0.0021
Observed species	196.6 ± 20.4 ^a^	177.2 ± 8.8 ^a^	136.2 ± 14.3 ^b^	0.0068
Shannon	5.2 ± 0.5	4.6 ± 0.5	4.3 ± 0.6	0.07

Note: the values are the mean plus standard deviation; CON = control, LCA = low cocktail of antibiotics concentration; LSA = low concentration of single antibiotic. Means with different superscript are significantly different (*P* < 0.05).

## Supplementary Materials

The following are available online at http://www.mdpi.com/2076-2607/6/4/118/s1.

## Abbreviations

MR	Milk Re-placer
CON	Control
LCA	Low cocktail of antibiotics concentration
LSA	Low concentration of single antibiotic
QIIME	Quantitative Insight Into Microbial Ecology
OTUs	Operational taxonomic units
LDA	Linear discriminant analysis
PCA	Principle component analysis
