# Supplementary material for: Low Concentration of Antibiotics Modulates Gut Microbiota at Different Levels in Pre-Weaning Dairy Calves
Source: Microorganisms. 2018 Nov 27;6(4):118. doi: 10.3390/microorganisms6040118 (PMC6313529; doi:10.3390/microorganisms6040118)
Supplement: Supplementary file 1 [file microorganisms-06-00118-s001.pdf]

Supplementary Materials

Table S1: The median concentration of Penicillin, streptomycin, tetracycline and ceftiofur residues in waste milk.

	**Penicillin**	**Streptomycin**	**Tetracycline**	**Ceftiofur**
Mean ± standard deviation (mg/L)	0.024 ± 0.034	0.019 ± 0.008	0.08 ± 0.05	0.76 ± 0.43
Median (mg/L)	0.024	0.025	0.10	0.33

Table S2: Description of the relative abundance of the predominant phyla and genera of the ileum, colon and rectum samples.

Ileum–Phylum	Genus	CON	LCA	LSA	P value
Firmicutes		0.709±0.08	0.620±0.18	0.590±0.23	0.63
	Acetitomaculum	0.016±0.02	0.146±0.14	0.023±0.02	0.10
	Romboutsia	0.093±0.08	0.013±0.02	0.157±0.30	0.54
	Bacillus	0.026±0.01	0.047±0.03	0.016±0.01	0.15
	Streptococcus	0.014±0.01	0.004±0.00	0.009±0.01	0.34
	Faecalibacterium	0.001±0.00	0.029±0.05	0.001±0.00	0.29
	Bacillus	0.0262±0.01	0.0473±0.03	0.0151±0.01	0.21
Proteobacteria		0.164±0.08	0.103±0.09	0.150±0.06	0.63
	Escherichia	0.101±0.07[a]	0.000±0.00[b]	0.019±0.03[ab]	0.02
	Comamonas	0.011±0.0[a]	0.009± 0.01[a]	0.054±0.03[b]	0.02
Actinobacteria		0.073±0.04	0.119±0.04	0.178±0.16	0.27
	Olsenella	0.054±0.04	0.106±0.04	0.153±0.16	0.39
Bacteroidetes		0.012±0.01	0.109±0.12	0.008±0.01	0.14
	Alloprevotella	0.003±0.00	0.022±0.03	0.001±0.00	0.29
	Bacteroides	0.001±0.00	0.009±0.01	0.009±0.00	0.17
Colon					
Firmicutes		0.584±0.19	0.652±0.20	0.640±0.16	0.82
	Faecalibacterium	0.184±0.09	0.294±0.14	0.241±0.14	0.59
	Blautia	0.044±0.00	0.084±0.04	0.070±0.01	0.13
	*Ruminococcus_gauvreauii_*	0.063±0.11	0.034±0.03	0.005±0.00	0.46
	Subdoligranulum	0.028±0.02	0.024±0.01	0.040±0.04	0.74
Bacteroidetes		0.348±0.22	0.307±0.20	0.277±0.14	0.84
	Alloprevotella	0.230±0.15	0.199±0.17	0.004±0.00	0.08
	Prevotellaceae_Ga6A1_ group	0.011±0.02[a]	0.012±0.01[a]	0.074±0.06[b]	0.02
	Bacteroides	0.030±0.03	0.037±0.03	0.137±0.10	0.13
Actinobacteria		0.041±0.04	0.024±0.02	0.076±0.05	0.29
Proteobacteria		0.007±0.01	0.006±0.01	0.004±0.00	0.81
Rectum					
Firmicutes		0.509±0.14	0.553±0.15	0.557±0.20	0.91
	Blautia	0.042±0.01	0.100±0.06	0.070±0.05	0.36
	Faecalibacterium	0.113±0.09	0.223±0.07	0.212±.19	0.54
	Eubacterium	0.049±0.04	0.027±0.00	0.049±0.06	0.36
	Megamonas	0.047±0.04	0.002±0.00	0.050±0.09	0.30
	phascolarctobacterium	0.036±0.02[a]	0.004±0.01[b]	0.005±0.00[b]	0.02
Bacteroidetes		0.401±0.19	0.407±0.16	0.399±0.23	1.00
	Alloprevotella	0.195±0.09[ab]	0.224±0.17[a]	0.010±0.01[b]	0.03
	Bacteroides	0.060±0.03	0.077±0.05	0.185±0.14	0.19
Actinobacteria		0.055±0.07	0.015±0.01	0.036±0.04	0.59
Proteobacteria		0.012±0.01	0.007±0.01	0.004±0.00	0.23

Note: The values are the mean plus SD; CON = control, LCA = low cocktail of antibiotics concentration; LSA = low concentration of single antibiotic. Means with different superscript are significantly different (P < 0.05).

Table S3: Description of the relative abundance of the predominant families of the ileum, colon and rectum samples.

Ileum–Family	CON	LCA	LSA	P value
Lachnospiraceae	0.2343±0.12	0.2717±0.15	0.1164±0.06	0.32
Peptostreptococaceae	0.1024±0.08	0.0155±0.02	0.1775±0.34	0.58
Ruminococcaceae	0.0480±0.02	0.0981±0.10	0.0330±0.01	0.36
Enterobateriaceae	0.1019±0.07a	0.0023±0.00b	0.0204±0.03ab	0.02
Coriobacteriaceae	0.0648±0.04	0.1128±0.04	0.1589±0.16	0.34
Colon				
Lachnospiraceae	0.1982±0.14	0.1835±0.07	0.1407±0.05	0.58
Ruminococcaceae	0.2962±0.11	0.4357±0.15	0.4203±0.14	0.37
Prevotellaceae	0.2969±0.18	0.2495±0.18	0.1129±0.05	0.10
Bacteroidaceae	0.0302±0.03	0.0368±0.03	0.1370±0.10	0.13
Coriobacteriaceae	0.0263±0.02	0.0223±0.02	0.0684±0.05	0.26
Rectum				
Lachnospiraceae	0.1189±0.05	0.1831±0.09	0.1134±0.09	0.50
Ruminococcaceae	0.2580±0.10	0.3395±0.08	0.3694±0.14	0.43
Prevotellaceae	0.2805±0.15	0.2977±0.15	0.1291±0.07	0.11
Bacteroidaceae	0.0598±0.03	0.0771±0.05	0.1847±0.13	0.19
Porphyromonadaceae	0.0282±0.01	0.0158±0.01	0.0577±0.10	0.32

Note: The values are the mean plus SD; CON = control, LCA = low cocktail of antibiotics concentration; LSA = low concentration of single antibiotic. Means with different superscript are significantly different (P < 0.05).

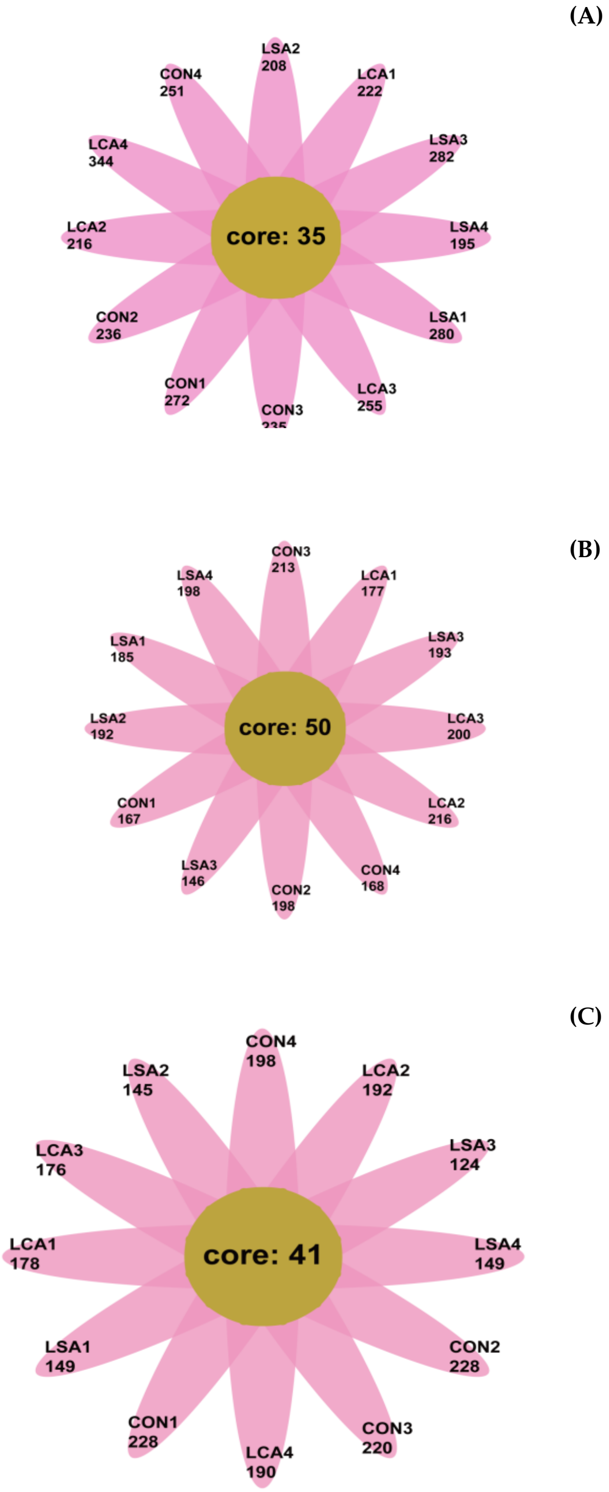

Figure (S1): The yellow circle represents the shared OTUs among animals in each treatments and the pink color represents number of OTUs in each individual. A= Ileum; B= colon and C= Rectum. CON= control; LCA= low cocktail of antibiotics concentration; LSA= low concentration of single antibiotic

(A)

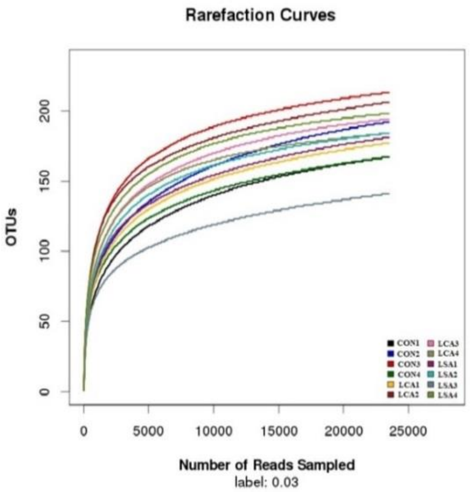

(B)

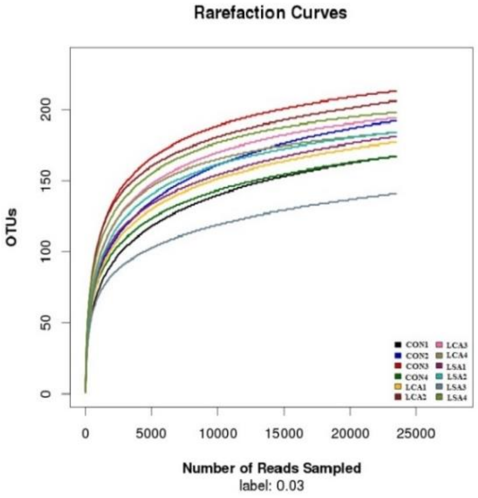

(C)

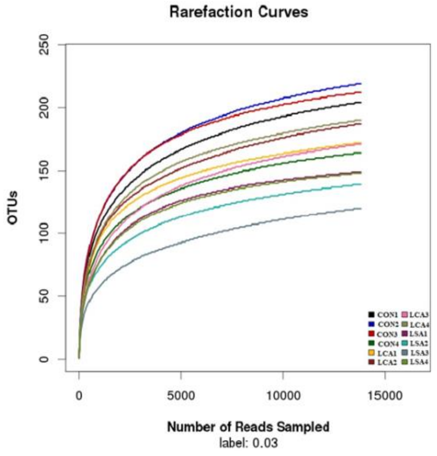

Figure (S2): Rarefaction curves of the microbial communities of ileum, colon and rectum samples where each curve represents one animal. A= Ileum; B= colon and C= Rectum. CON= control; LCA= low cocktail of antibiotics concentration; LSA= low concentration of single antibiotic. Each curve represents one individual sample.
